# Pozzolanic Activity of Zeolites: The Role of Si/Al Ratio

**DOI:** 10.3390/ma12244231

**Published:** 2019-12-17

**Authors:** Barbara Liguori, Paolo Aprea, Bruno de Gennaro, Fabio Iucolano, Abner Colella, Domenico Caputo

**Affiliations:** 1 Dipartimento di Ingegneria Chimica, dei Materiali e della Produzione Industriale, Università Federico II di Napoli, Piazzale V. Tecchio 80, 80125 Naples, Italy; paolo.aprea@unina.it (P.A.); bruno.degennaro@unina.it (B.d.G.); fabio.iucolano@unina.it (F.I.); domenico.caputo@unina.it (D.C.); 2 Dipartimento di Scienze della Terra, dell’Ambiente e delle Risorse, Università Federico II di Napoli, Complesso di Monte Sant’Angelo (Edificio L), Via Cinthia, 21-80126 Naples, Italy; abner.colella@unina.it

**Keywords:** FAU-type zeolites, pozzolanic activity, Si/Al ratio, thermogravimetry

## Abstract

A great challenge of research is the utilization of natural or synthetic zeolites, in place of natural pozzolans, for manufacturing blended cements. The difficulties of interpretation of the pozzolanic behavior of natural zeolite-rich materials and the role played by their nature and composition can be overcome by studying more simple systems, such as pure synthetic zeolites. This study aims at investigating the pozzolanic ability of isostructural zeolites with different framework compositions, such as three sodium zeolites of the faujasite (FAU) framework type: LSX, X, and Y. The pozzolanic activity has been estimated by thermogravimetry and X-ray diffraction analysis. The overall outcome of the investigation is that the zeolite structure affects its pozzolanic activity, as zeolites with similar framework densities exhibit distinct abilities to fix lime. Moreover, the framework composition is effective either from a kinetic point of view or on the total amount of fixed lime. Zeolite X appears to possess the best average features.

## 1. Introduction

The raising attention to environmental problems (e.g., the worrying increase of CO_2_ discharges into the atmosphere) has resulted in a renewed interest in supplementary materials for the cement industry, particularly those that can act as pozzolana. A pozzolanic material is generally defined as a natural, synthetic, or secondary raw material, containing high percentages of active SiO_2_ and Al_2_O_3_. These can react, in the presence of water, with lime or lime-releasing materials (e.g., Portland clinker), giving products such as hydrated calcium aluminates and hydrated calcium silicates, having a hydraulic character, i.e., displaying mechanical resistances. Among the many pozzolanic materials, zeolites have attracted increasing interest in recent years.

Zeolites are crystalline microporous aluminosilicates, whose framework consists of SiO_4_ and AlO_4_ tetrahedra, joined together in various regular arrangements through the sharing of oxygen atoms. This results in open structures with crossing channels and cages, which are occupied by guest molecules (usually water) and extra-framework, poorly bonded, alkaline, or alkaline earth cations, balancing the negative charge of the AlO_4_ tetrahedra. The extensive inner surfaces and the resulting adsorption properties, combined with molecular sieving and cation exchange abilities, give these materials unique and valuable possibilities of application in dozens of processes of industrial relevance (see, e.g., [1,2,3,4]).

A great deal of research has been carried out on the utilization of either natural [5,6,7,8,9] or synthetic [10,11,12,13] zeolites, in place of natural pozzolans, for manufacturing blended cements and in the preparation of a sustainable binder [14,15]. The large specific surface area, featuring an intrinsic metastability (see, e.g., [16]), gives the zeolite a good pozzolanic activity. Recently, Molinari et al. [17] reviewed the applications of natural zeolites and modified clays in the construction industry, analyzing their role in the technological behavior of several building materials. Some synthetic zeolites–lime systems were also studied, analyzing the effect of zeolite addition on mechanical properties [18,19].

In a lime/zeolite system, different simultaneous reactions and equilibria occur, e.g., for a lime/Na–zeolite system: 

(a) Dissolution of solid (s) Ca(OH)_2_ and related dissociation equilibria:

(1) $$\mathrm{Ca}{\left(\mathrm{OH}\right)}_{2}\left(s\right)\;⇄\;\mathrm{Ca}{\left(\mathrm{OH}\right)}^{+}{+\;\mathrm{OH}}^{-}$$

(2) $$\mathrm{Ca}{\left(\mathrm{OH}\right)}^{+}\;⇄{\;\mathrm{Ca}}^{2+}{\;+\;\mathrm{OH}}^{-\;}.$$

(b) Ion–exchange equilibria involving Ca^2+^ and Ca(OH)^+^ in solution and Na^+^ in zeolite (z):

(3) $${2\mathrm{Na}}^{+}\left(z\right)+\;\mathrm{Ca}{\left(\mathrm{OH}\right)}^{+}⇄{\;\mathrm{Ca}}^{2+}\left(z\right){+\;2\mathrm{Na}}^{+}{+\;\mathrm{OH}}^{-}$$

(4) $${2\mathrm{Na}}^{+}\left(z\right){+\;\mathrm{Ca}}^{2+}⇄{\;2\mathrm{Na}}^{+}{+\;\mathrm{Ca}}^{2+}\left(z\right).$$

(c) Breakdown and dissolution of zeolite in basic solution and/or conversion into a transient amorphous material, followed by the precipitation of hydrated calcium aluminates (CAH) and hydrated calcium silicates (CSH). The aluminate and silicate magmas, rich in Ca^2+^, tend to give mixtures of silicates and aluminates, instead of aluminosilicates (framework silicates), as it happens in environments where the alkaline cations are predominant [20].

When a zeolitic material is involved in a pozzolanic reaction, different factors can affect its reactivity, namely: (a) structure of the zeolitic phase, (b) its specific surface, (c) framework (Si/Al) and extra-framework (exchangeable cations) composition, and (d) water/mix ratio.

Many papers have investigated the pozzolanic behavior of natural zeolite-rich materials and the role played by their nature and composition on the connected reaction mechanisms [21,22,23,24]. Mertens et al. [21] demonstrated that over longer time periods, the pozzolanic reaction is controlled by silica and alumina active contents, whereas the reactivity in the short term is affected by the cation content of the zeolites. However, these systems are rather complex, as in natural zeolites (i.e., zeolitic tuffs), several components normally co-exist, namely different zeolites, clay minerals, and crystalline or amorphous ancillary minerals. In addition, some of these phases exhibit pozzolanic properties (e.g., zeolites, clays, glass, slags, pumice, gel-like phases, etc.); others, due to their structural compactness and/or their chemical stability, are largely inert (e.g., crystalline silica, feldspars, pyroxene, biotite, etc.). However, the unavoidable difficulties of interpretation can be overcome by studying more simple systems, such as those in which pure, synthetic zeolites, better if commercially available, are considered. That is why in a previous paper [11], two synthetic zeolites, types A [LTA] and X [FAU], having close Si/Al ratios and sharing the same polyhedral building units have been studied. It has been demonstrated that zeolite A reacts more readily than zeolite X, but zeolite X, being slightly more siliceous, contributes to a greater extent to the development of the mechanical resistances of the cement pastes at short curing times.

An interesting challenge to provide useful indications on the use of zeolites in manufacturing pozzolanic cements can be completing the overall picture by focusing on the influence of the framework composition of synthetic isostructural zeolites with different Si/Al ratios. So, the present investigation focuses on the influence of framework composition on the pozzolanic activity of three different synthetic faujasite-type (FAU) [25] sodium zeolites, namely LSX (Si/Al ratio close to 1), X (~1.25), and Y (2.5–2.8), which have been selected as the pozzolanic addition in lime-based pastes. The reaction mechanism between zeolite and lime has been investigated by comparing the results of thermogravimetric and X-ray diffraction analyses, the former giving an account of the kinetics of lime disappearance, and the latter monitoring both zeolite disappearance and the formation/evolution of the reaction products.

## 2. Materials and Methods

### 2.1. Materials

The more siliceous X and Y, two Na zeolites belonging to the FAU framework type [25], are synthetic, commercial products. In particular, the species X (formally Molecular Sieves 13X) is marketed by Carlo Erba AnalytiCals (Italy), while the species Y (labeled Y–54) is a product of UOP (Molecular Sieve Division).

The less siliceous LSX, namely “low silica X”, containing an equal number of tetrahedral Al and Si atoms within the framework and having therefore the most regular Si–Al distribution in the faujasite series, was a synthesis product, which was obtained according to the method developed by Kühl [26]. Crystallization was achieved starting from sol–gel mixtures having the following molar composition: SiO_2_/Al_2_O_3_ = 2.2, (Na_2_O+K_2_O)/SiO_2_ = 3.58, Na_2_O/(Na_2_O+K_2_O) = 0.77, and H_2_O/(Na_2_O+K_2_O) = 13.68. More specifically, sodium and potassium hydroxides (products of Carlo Erba AnalytiCals) and then sodium silicate were gradually added to an aqueous solution of sodium aluminate (both chemicals from Sigma Aldrich). Aging without stirring was scheduled at 70 °C for 3 h; then, the temperature was raised to 100 °C for 2 h until complete crystallization. After cooling to room temperature, the solid was filtered, rinsed with bidistilled water, and dried at 60 °C overnight. Finally, before any further use, the obtained LSX sample, containing both Na^+^ and K^+^, was exhaustively exchanged in sodium form through repeated contacts with fresh NaCl solutions. The obtained product will be indicated from here on as L.

Each zeolite sample was calcined and digested in acid solution by microwave-induced heating (Perkin–Elmer Multiwave 3000 oven) to assess the chemical composition. Fluoride complexation was attained by adding boric acid. Cationic concentration was analyzed in the resulting solution by ICP–OES (Inductively Coupled Plasma- Optical Emission Spectroscopy, Perkin–Elmer Optima 2100 DV).

SEM observations were carried out with a Cambridge S440 instrument. The grain size distribution of the zeolite samples was estimated through an SEM image analysis with the help of the ImageJ processing program. An area of about 2000 μm has been sampled for each zeolite.

Specific surface areas (Brunauer Emmet Teller (BET) method) of all the samples were measured at 77 K using a Micromeritics ASAP 2020 instrument.

The thermogravimetric (TG) profile of the investigated materials was obtained with a Netzsch STA409 PCLuxx apparatus (alumina crucibles; N_2_ gas flow; heating rate: 10 °C/min) in the temperature range of 25–1000 °C.

A commercial sample of lime was utilized in all experiments. Its composition, estimated by TG analysis, was as follows: Ca(OH)_2_ 88.5%, CaCO_3_ 5.7% plus impurities. 

All the XRD analyses were performed using a X’Pert Pro (PANalytical) diffractometer (Cu Kα radiation, 2θ range: 5–60°, step width: 0.02° 2θ; scan speed: 0.02° 2θ/s; slit width: 0.5°).

### 2.2. Estimation of Pozzolanic Activity

Several methods have been proposed to estimate the pozzolanicity, i.e., the pozzolanic ability, of a material. The official test, called Fratini’s test, which is recognized by the European Standards [27], involves a hydrothermal treatment of a standardized mortars, made of Portland clinker and gypsum plus the pozzolanic material under investigation. The effectiveness of the pozzolanic action is proved by Ca^2+^ and OH^−^ concentration in solution at the end of the treatment, since it is an indirect sign of the presence/absence of “free lime” in the solid.

In agreement with the prescribed procedure, 20 g of each experimental blend (zeolite/Portland clinker weight ratio equal to 1 + gypsum) were mixed with 100 ml of deionized water and kept at 40 °C for 8 days or 15 days (if the test was negative after 8 days). Ca^2+^ and OH^−^ concentrations were estimated in the mother liquor at the end of the experiment through the ordinary methods of volumetric analysis. Results (average values of runs performed in triplicate) were projected in a plot, reporting the solubility curve at 40 °C of Ca(OH)_2_ (expressed as CaO) as a function of OH^−^ concentration in solution. Comparing the experimental data with the equilibrium value provides an indication of the pozzolanic ability of the selected sample.

Other procedures, which are available from the literature and based on the direct estimation of Ca(OH)_2_ amount reacted in the studied system, can be used for monitoring the pozzolanic action of the investigated material [28,29].

To measure the free lime content in the pastes at different curing times, several lime–zeolite blends, containing equal weighted amounts of the two components (5 g + 5 g), were carefully prepared. Then, a suitable amount of distilled water was added to the blends to obtain a good workability of the resulting pastes. After a careful mixing, pastes were transferred in sealed polyethylene molds and kept at room temperature for different curing times. All the above operations were carried out very readily and avoiding the contact with air, in order to contrast the possible formation of CaCO_3_. That is why the small amount of calcium carbonate that was found at the end of each experiment may be considered as an impurity of the parent hydroxide (see the Experimental section).

At programmed times, from 3 hours to 90 days, solidified pastes were collected, ground, treated with acetone to stop further hydration, and lastly stored in sealed containers until analysis.

The estimation of lime amount in the pastes was performed by TG analysis (see the previous section).

Typically, a hydraulic mortar, made of lime and a natural pozzolan, shows three endothermic effects with associated weight losses:

(a) at about 150 °C, due to CAH and CSH dehydration;

(b) in the range of 400–550 °C roughly, due to Ca(OH)_2_ dehydroxylation with water removal;

(c) in the range of 600–800 °C due to CaCO_3_ decarbonation with CO_2_ removal.

Therefore, the weight loss measured within 200 °C is an indirect indication of the formation of compounds having a hydraulic character, whereas the amount of reacted Ca(OH)_2_, which was calculated from the water amount released from 350 to 550 °C, is an indirect measure of the advancement of the pozzolanic reaction. When the pozzolanic material is a zeolite, difficulties arise in the interpretation of TG profiles, because of the joint presence of zeolitic water. It is well known that zeolite presents several physical and chemical changes on heating [30]. Among the others, the zeolitic water, occupying different sites within the framework, smoothly leaves from the lattice in a wide thermal range, usually from room temperature to roughly 300 °C (although a minor but usually negligible water loss due to the presence of a few hydroxyls in zeolite structure sometimes occurring). The total amount of released water, measured by thermogravimetry (TG), and the position and shape of the endotherms, highlighted in DTA traces, are functions of different factors, such as the zeolite type and its chemical composition.

For the above reasons in lime–zeolite pastes or mortars, the formation/evolution of the CAH and CSH cannot be monitored by the thermograms, since the dehydration of the above phases is hidden by the loss of zeolitic water. To overcome this difficulty, a supplementary technique, XRD analysis, was used to monitor the disappearance of zeolites and the formation/evolution of the hydration products.

## 3. Results and Discussion

### 3.1. Materials Characterization

The three FAU-type zeolites, named in the following as LSX, X, and Y, were carefully characterized before testing them in pozzolanic activity experiments. In order to avoid the influence of the extra-framework cations, the LSX sample was exhaustively exchanged in sodium form before any further use. The obtained product will be indicated from here on as L.

Table 1 reports the chemical composition of the samples together with some of their physical features.

The chemical data appear to be intrinsically congruent and in agreement with those reported in the literature. In particular, the relationship between the Si/Al ratio and the cell parameter fits the empirical correlation found for the species X and Y [31], whereas it substantially fails for the species L, because its framework composition falls outside the range considered by these authors. On the other hand, it is worth noting that the exchange procedure resulted in a decrease in the Si/Al ratio, which was due to a probable leaching of silica. Moreover, it was impossible to remove all K^+^ from the original LSX framework, notwithstanding the repeated contacts with fresh NaCl solutions.

**Figure 1:**
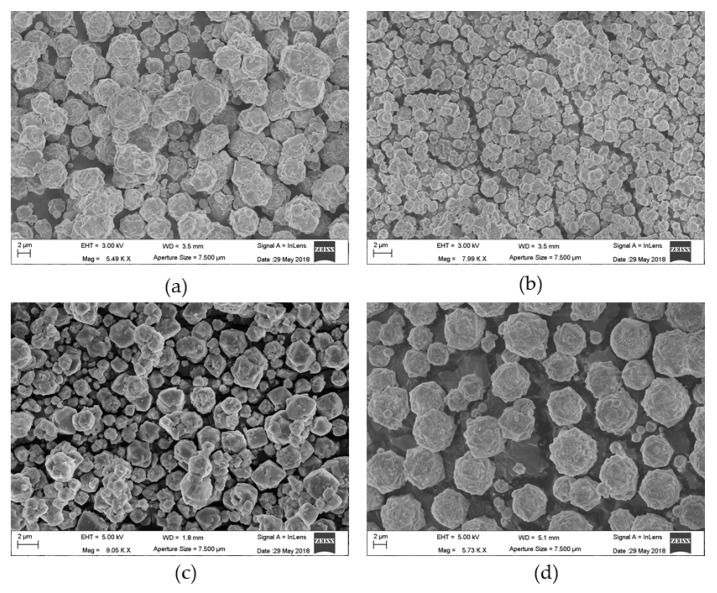
Scanning electron micrographs of (**a**) X, (**b**) L, (**c**) Y, and (**d**) LSX zeolites.

Figure 1 shows the micrographs of the investigated samples. The samples appear as aggregates of microcrystals having a round morphology, although the cubic habit of the crystals is easily recognizable. The grain dimensions are different from one sample to another and even within the same sample.

Table 2 shows the results of the grain size analysis. Data, referring to a population of 60 to 90 particles detected over an area of 2000 μm, show a relevant grain size difference of the three sodium zeolites (L, X, and Y), whereas the values are rather homogenous within the same sample (average and median values very close to each other). 

**Figure 2:**
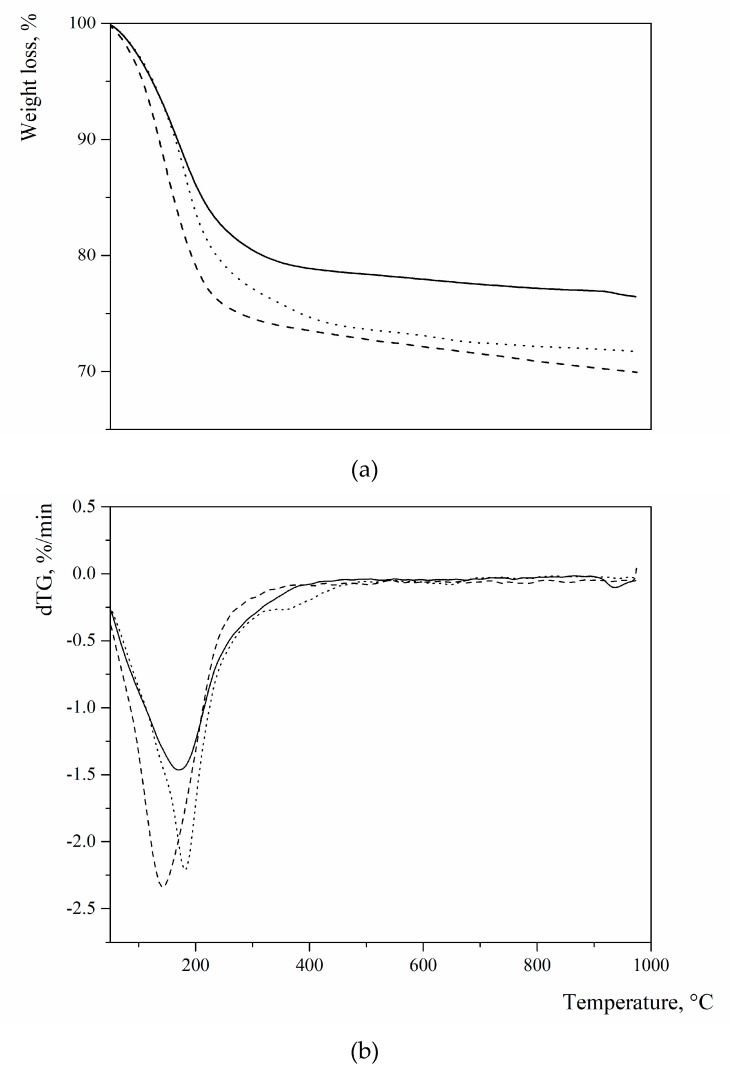
Thermogravimetry (TG) (**a**) and (**b**) dTG profiles of the three zeolites used as pozzolanic additions (pointed line = zeolite L; continuous line = zeolite X; dashed line = zeolite Y) (operation parameters: alumina crucibles; N_2_ gas flow; heating rate: 10 °C/min; temperature range 25–1000 °C).

Figure 2 summarizes the thermal profiles of the three sodium FAU-type zeolites, which were detected by thermogravimetry (TG). As evidenced by the derivative of the TG, all of them present a broad endothermic effect in the temperature range of –300 °C with minima at 183, 170, and 143 °C for L, X, and Y, respectively, due to the loss of “zeolitic” water, which was present in the cavities of the zeolite structure and bonded to extra framework cations.

**Figure 3:**
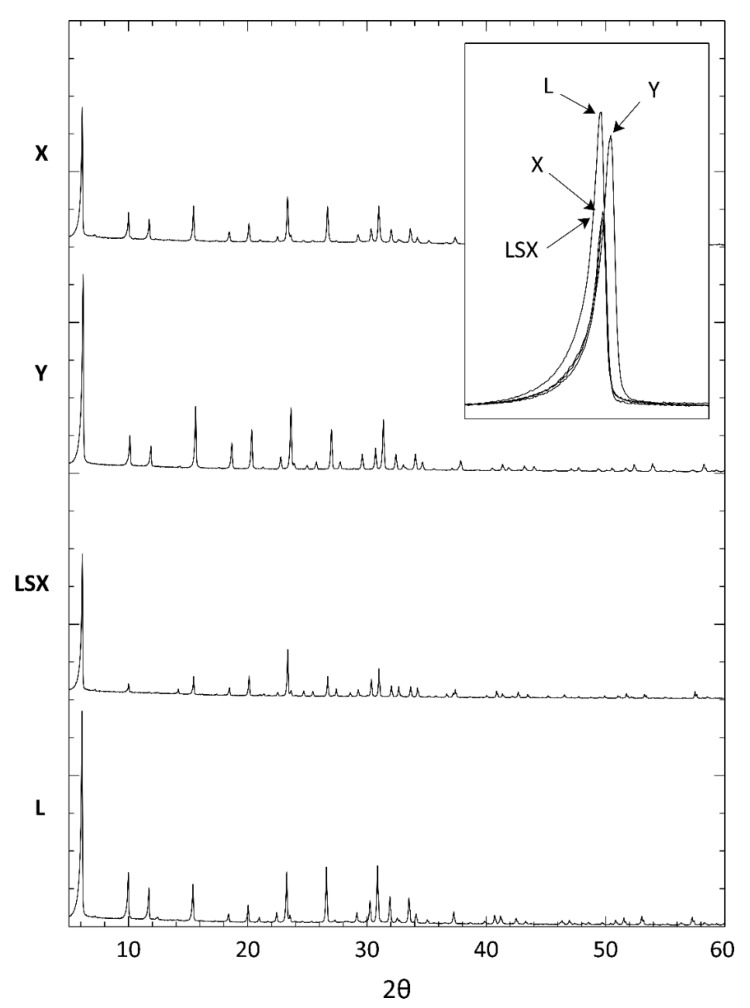
XRD patterns of the faujasite (FAU)-type zeolites. The inset highlights the shifts in the main peak position.

Figure 3 reports the XRD patterns of the four FAU-type zeolites. The patterns do not present any additional reflections due to impurities and are identical to those of the reference materials, which were published by the International Centre for Diffraction Data: LSX (ICDD PDF#89–0769), NaX (ICDD PDF#72–2422), and Na–Y (ICDD PDF#43–168) for LSX, X, and Y, respectively. The four spectra are practically coincident with each other, apart from some small shifts of the corresponding reflections and some differences in the peak intensities (see the inset in Figure 3). The values of the cell parameter calculated by indexing the reflections in the cubic system (space group *Fd–3m*) [25] are reported in Table 1.

### 3.2. Preliminary Evaluation of Pozzolanicity

**Figure 4:**
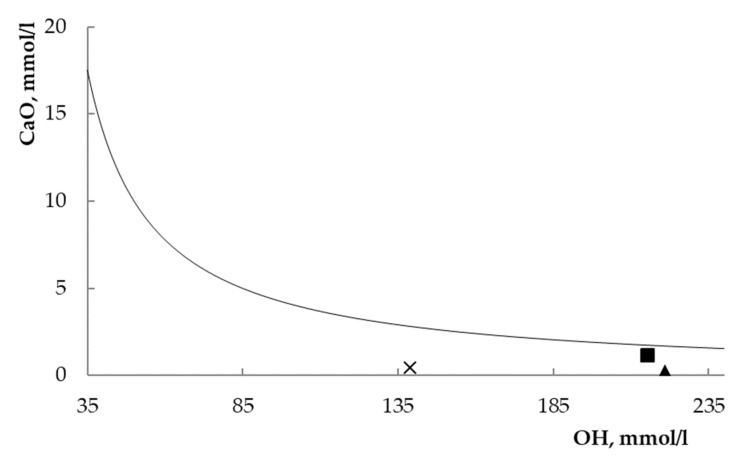
Fratini’s test [27] for the three investigated systems after eight-day contact. square = zeolite L; triangle = zeolite X; cross = zeolite Y.

The estimation of pozzolanicity, i.e., the pozzolanic ability, was performed by the official test, called the Fratini test, recognized by the European Standards. Figure 4 shows the results of the Fratini test. In this plot, points over the curve or on the curve are representative of oversaturated or saturated solutions (the absence or deficiency of pozzolanic activity); on the contrary, points under the curve are representative of undersaturated solutions (the presence of pozzolanic activity). The points representative of the three sodium FAU zeolites fall under the solubility curve of Ca(OH)_2_. Therefore, the three materials confirm their pozzolanic ability.

A quantitative evaluation may also be worked out from the Fratini plot. The UNI EN196–5 standard [27] provides, in fact, the solubility data of Ca(OH)_2_ (as CaO) at 40 °C in the 35–90 mmol/l [OH^−^] range, under the following mathematical formulation:

(5) $${\left[CaO\right]}_{eq}=\frac{350}{\left[OH^{-}\right]-15}.$$

Table 3 summarizes the analytical data calculated from the data of Figure 4 with the help of Equation (5). Inspecting both Figure 4 and Table 3 points out that zeolites X and Y result in an unsaturated degree of the system much higher than zeolite L, and therefore, they are more effective as pozzolanic materials than the less siliceous FAU zeolite.

### 3.3. Reactions in Zeolite–Lime–Water System

**Figure 5:**
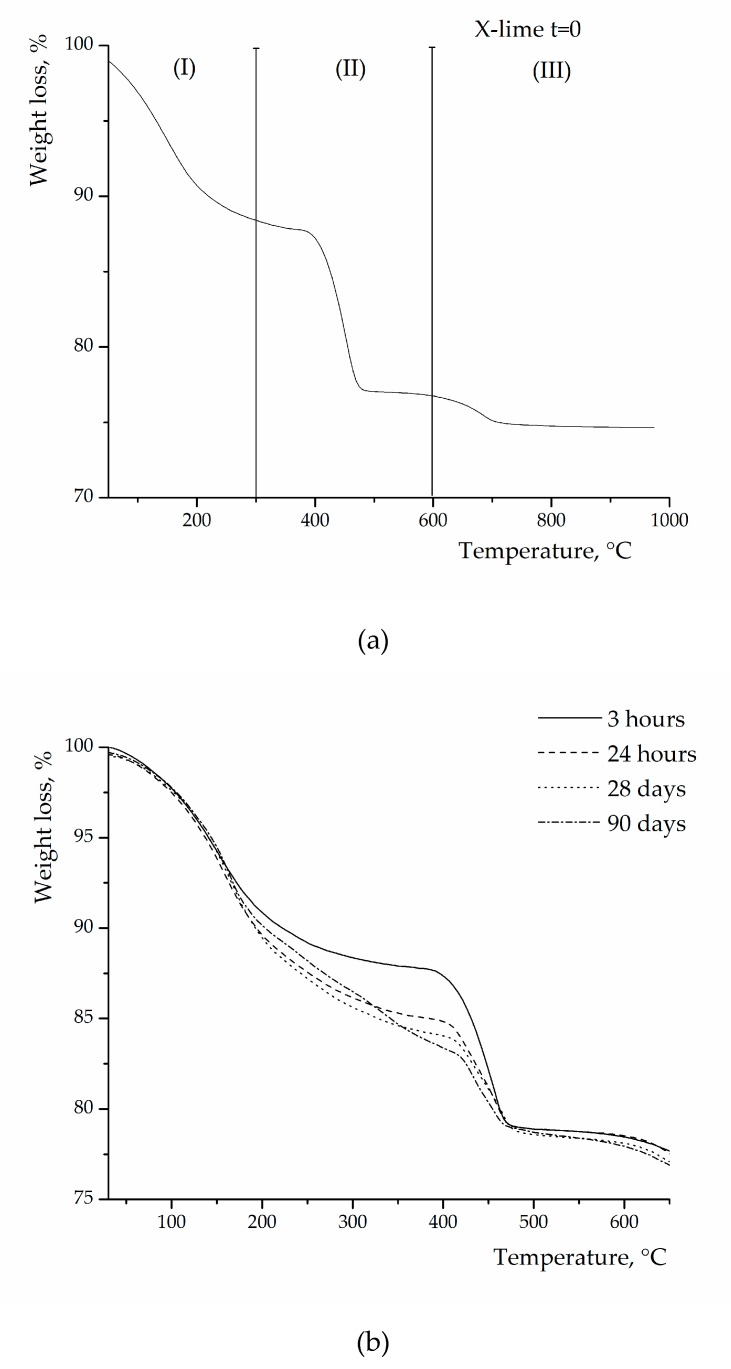
Thermograms of zeolite X–lime blends before the beginning of reaction (**a**) and at different curing times (**b**).

The progress of the pozzolanic reaction between lime and zeolites has been evaluated by measuring the free lime content in the pastes at different curing times. Figure 5a shows a typical thermogram of a zeolite–Ca(OH)_2_ blend at t = 0, i.e., before the addition of water and therefore before the pozzolanic reaction starts. The zeolite is in this case zeolite X. Three major thermal effects are observed in the curve: (i) the weight loss in the region I (100–300 °C) is connected to the release of “zeolitic” water; (ii) the weight loss in the region II (350–550 °C) is attributed to the dehydration of Ca(OH)_2_; and (iii) the small weight loss in the region III (550–850 °C) is attributed to the decarbonation of CaCO_3_ (originally present in the parent hydroxide).

After water addition, upon increasing the curing time (t > 0), regardless of the zeolite type, a progressive decrease of lime content in the pastes is observed due to the progress of the reaction between silicoaluminate species, coming from zeolites, and lime, to form calcium silicate hydrate (CSH) and calcium aluminate hydrate (CAH). Figure 5b shows a series of thermograms, still concerning zeolite X, obtained at various reaction times for zeolite–Ca(OH)_2_ pastes. Note that the presence of zeolite hides, especially in the early stages of the reaction, the possible dehydration effects due to CSH and CAH. Analogous curves, as those in Figure 5, were obtained for the pastes made with the other two zeolites.

The estimation of lime amount in the pastes was performed by thermogravimetric analysis, using the following equation:

(6) $$m_{L\left(r\right)}\%\;=\frac{m_{L\left(i\right)}\;-\;4{.11m}_{w}}{m_{L\left(i\right)}}\times 100$$

where m_L(r)_ and m_L(i)_ are the reacted and initial amounts of lime in the paste, respectively, m_w_ is the amount of released water, and 4.11 is the stoichiometric conversion factor from water to lime in the reaction of lime dehydroxylation. 

Table 4 collects all the data concerning Ca(OH)_2_ dehydroxylation. Calculated data, concerning the residual unreacted lime, were estimated from the region II of the various thermograms and processed with Equation (6).

At short curing times (3 hours), the zeolites showed different behaviors: the less siliceous L and X fixed roughly 22% and 18% of the initial lime amount, respectively, whereas the most siliceous Y reacted with only 8% of lime. As reported in the introduction, the first stage of the whole pozzolanic reaction in a zeolite–lime system consists of cation exchange, which is a fake pozzolanic reaction, because Ca^2+^ is removed but not fixed. Accordingly, zeolites L and X, having a higher aluminium content and therefore a higher cation exchange capacity, resulted as more effective in subtracting Ca^2+^ from the system compared to Y, which was in accordance with the data reported by Mertens et al. [21]. It should be noted that this behavior may also depend at least in part on the smaller average grain size of zeolites L and X compared to Y (see Table 2). 

Zeolite L–lime pastes reached the maximum amount of fixed lime (around 30%) after 3 days, whereas zeolites X kept reacting until 28 days, fixing roughly 44% of Ca(OH)_2_ and Y still longer, fixing some 47% of lime after 90 days. After the first stages, in fact, where cation exchange prevails, both the higher specific surface of the zeolite and its higher silica-to-alumina ratio (see Table 1) become the key factors in the progress of the pozzolanic reaction [11,21].

**Figure 6:**
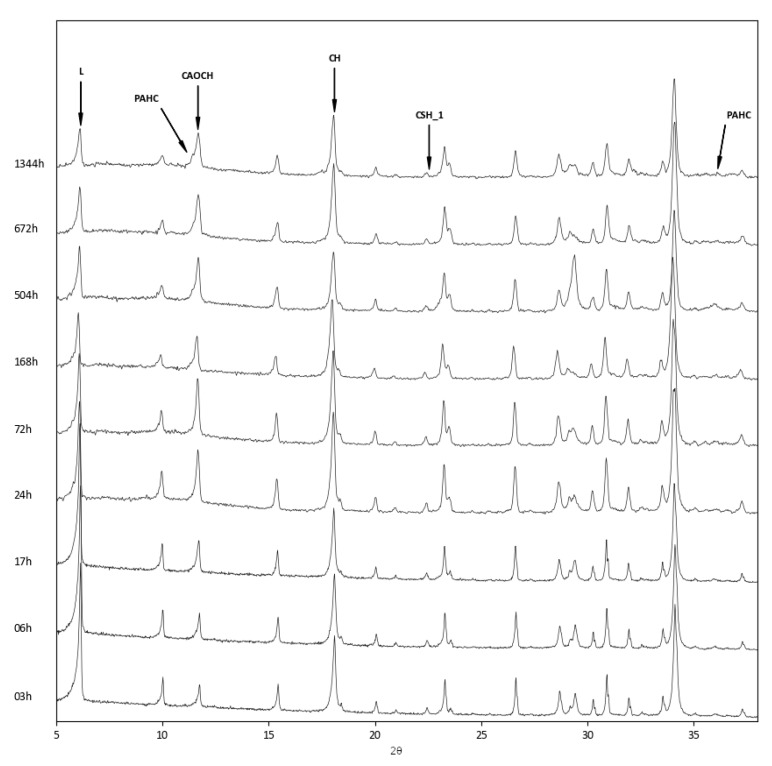
XRD patterns of zeolite L–lime–water system at different curing times. L = Na–enriched zeolite LSX; PAHC = paraalumohydrocalcite; CAOCH = calcium aluminum oxide carbonate hydrate; CH = calcium hydroxide; CSH_1 = calcium silicate hemihydrate.

**Figure 7:**
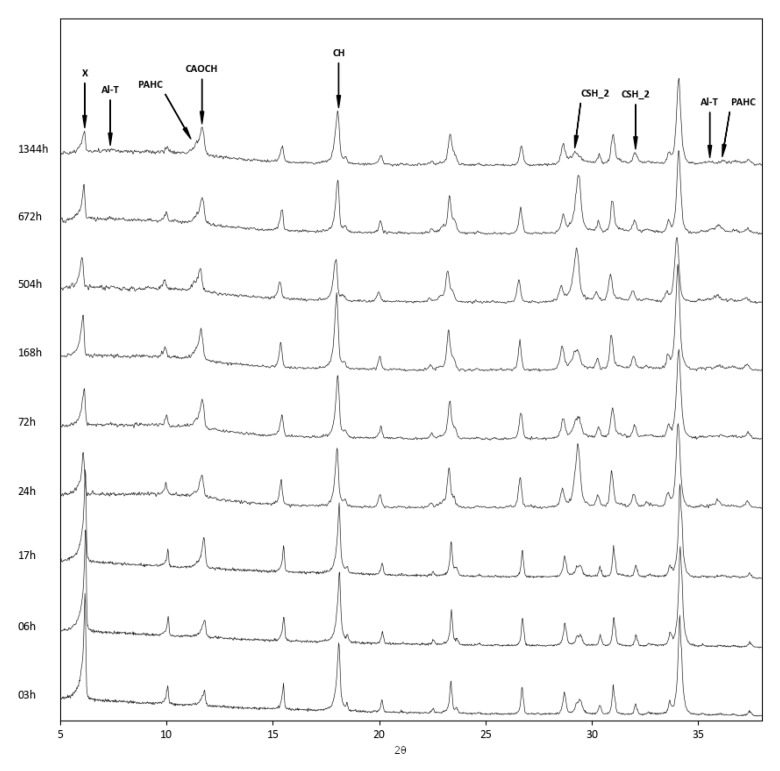
XRD patterns of zeolite X–lime–water system at different curing times. X = zeolite X; Al–T = aluminium tobermorite; PAHC = paraalumohydrocalcite; CAOCH = calcium aluminum oxide carbonate hydrate; CH = calcium hydroxide; CSH_2 = calcium silicate monohydrate.

**Figure 8:**
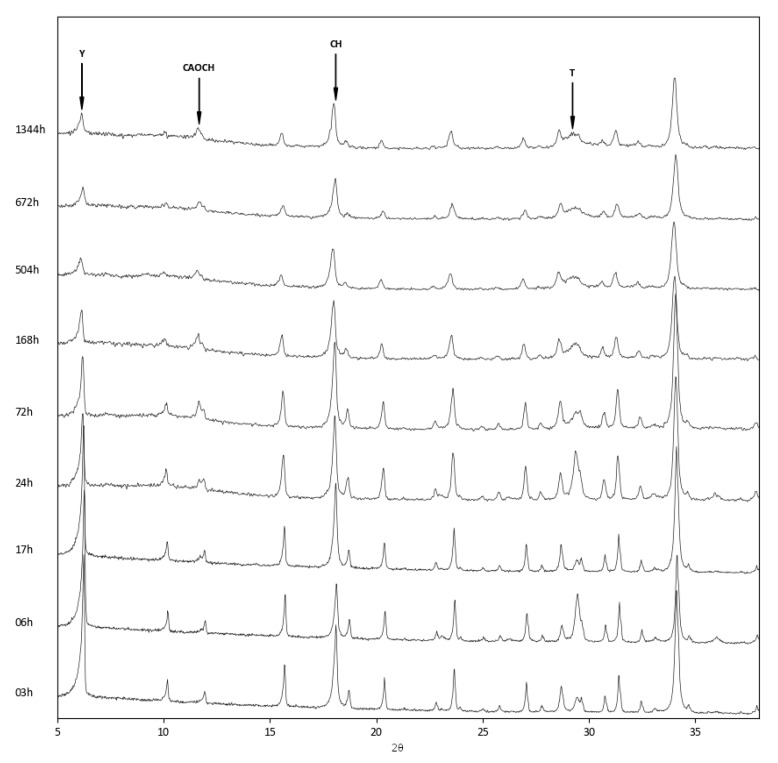
XRD patterns of zeolite Y–lime–water system at different curing times. Y = zeolite Y; CAOCH = calcium aluminum oxide carbonate hydrate; CH = calcium hydroxide; T = tobermorite.

The above findings were confirmed by the XRD analysis of the three zeolite–lime systems cured at different times (Figure 6, Figure 7 and Figure 8). In all the investigated systems, the specific zeolite, calcium hydroxide, and calcium carbonate were always detectable, although in different relative proportions, depending on the reaction times. 

The appearance of a calcium aluminum oxide carbonate hydrate (3CaO·Al_2_O_3_·CaCO_3_·11H_2_O, ICDD ref. code: 00–041–0219) was detected in all three systems. This product originates from the carbonation of calcium aluminate hydrate, which is the first to form in the reaction between zeolite and lime.

The inspection of the XRD patterns of the zeolite L–lime system revealed the presence of paraalumohydrocalcite (CaAl_2_(CO_3_)_2_(OH)_4_·6H_2_O, ICDD ref. code: 00–030–0222), together with traces of calcium silicate hemihydrate (Ca_2_SiO_4_·0.5H_2_O, ICDD ref. code: 00–015–0642) (Figure 6). 

In the zeolite X–lime system, the following hydrated phases were detected: paraalumohydrocalcite, traces of aluminum tobermorite (Ca_5_Si_5_Al(OH)O_17_·5H_2_O, ICDD ref. code: 00–041–0219), and calcium silicate monohydrate (CaO·SiO_2_·H_2_O, ICDD ref. code: 00–034–0002) (Figure 7).

Lastly, in the zeolite Y–lime system, the presence of a tobermorite-like phase (Ca_5_Si_6_O_16_(OH)_2_·8H_2_O, ICDD ref. code: 00–029–0331) (Figure 8) was observed.

### 3.4. Reaction Kinetics

The data of TG analysis (lime dehydroxylation) were further processed to investigate the mechanism of the lime–pozzolan reaction. It was assumed that the process of CSH and CAH formation, governed by the interaction between zeolite and the ionic calcium species (see Equations (1) and (2)), can be described by a second-order kinetic equation:

(7) $$L_{\left(t\right)}\;=\frac{{\mathrm{kL}}_{e}^{2}}{1+kL_{e}t}t$$

in which L_(t)_ and L_e_ are the fixed lime percentages at time t and at equilibrium, respectively, and k (w% h^−1^) is the rate constant of the reaction. Such reaction can be linearized as follows:

(8) $$\frac{t}{L_{\left(t\right)}}=\frac{1}{{\mathrm{kL}}_{e}^{2}}+\frac{t}{L_{e}}.$$

**Figure 9:**
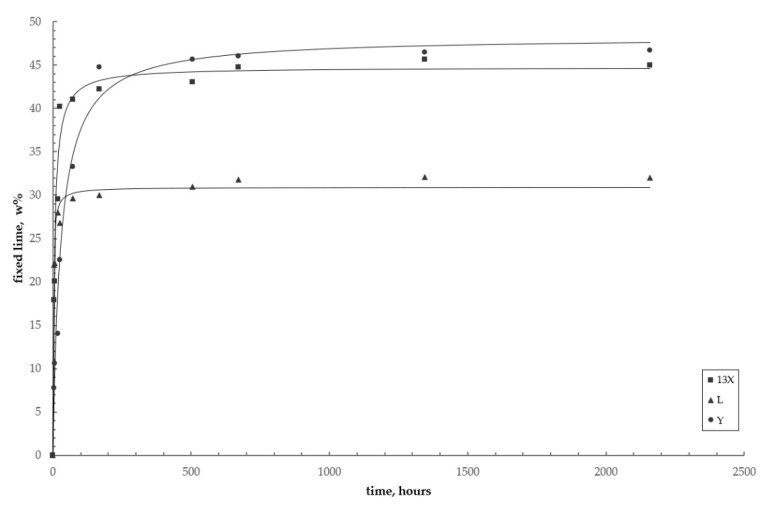
Reaction kinetics of the pozzolanic reaction for the three investigated systems. Circles = zeolite Y; squares = zeolite X; triangles = zeolite L. Curves = pseudo-second-order kinetic model (Equation (7)).

Figure 9 shows the kinetic curves of the pozzolanic reaction for the three zeolites considered. Data, corresponding to those of Table 4, are interpolated with curves derived from the kinetic model (Equation (7)).

**Figure 10:**
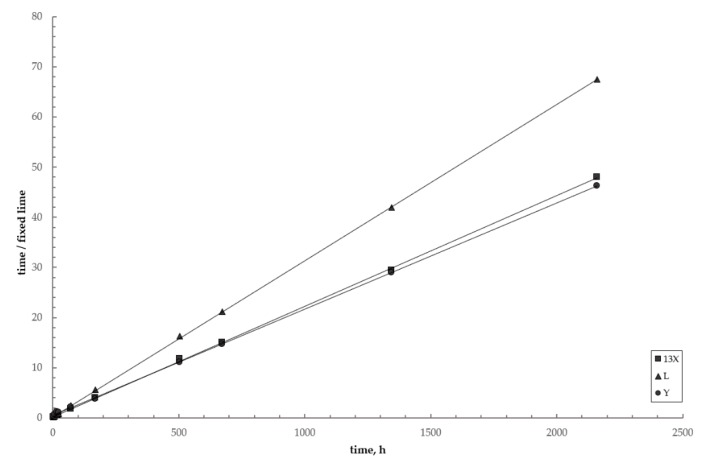
Linear plots for the three investigated systems. Circles = zeolite Y; squares = zeolite X; triangles = zeolite L. Lines = pseudo-second-order kinetic model (Equation (8)).

Figure 10 shows the linearized plots for the three zeolite–lime–water investigated systems (Equation (8)). The plots are well fitted by straight lines, such confirming the excellent agreement between the data and the model. Table 5 collects the corresponding best fitting parameters.

An inspection of Table 5 and Figure 9 shows that:

(i) The systems with the two less siliceous zeolites reach the equilibrium values rather rapidly (after some 7 days for zeolite L and after 28 days for zeolite X), as the experimental data are very close to the equilibrium data, which were obtained theoretically from the model (second column in Table 5), whereas the trend of the curve corresponding to the more siliceous system is still increasing after 90 days;

(ii) The zeolite L–lime–water system has the fastest reaction kinetics, while the zeolite Y–lime–water system has the slowest one;

(iii) On the contrary, zeolite L appears to be the least effective in subtracting Ca(OH)_2_ from the system, compared to the more siliceous X and Y, as already observed in the preliminary pozzolanicity test, which was carried out according with the European standards (see Figure 4 and Table 3).

Comparing these evidences with our previous results, it appears that the two main parameters examined, the structure and framework composition, i.e., siliceousness, act in a distinct way. It is generally known that zeolites react as more readily as their framework densities are lower, but it has been demonstrated that each structure with their structural peculiarities can diversely affect the reaction kinetics. In fact, the framework densities of LTA and FAU are very close to each other, 12.9 and 12.7 T/1000 Å^3^ (where T stands for tetrahedra), respectively, but the LTA framework appears to be more easily attacked by an alkaline, Ca^2+^-rich solution than a FAU framework [11]. On the other hand, the prevalence of silicon over aluminium in the framework, although negatively affecting zeolite activity in the early stages of the reaction, enables fixing a greater amount of calcium at longer curing times, therefore exhibiting a better pozzolanic activity.

The best compromise appears to be zeolite X, which exhibits a reasonable reaction rate in the early stages of the reaction together with a good effectiveness at fixing lime at longer curing times.

## 4. Conclusions

The results of this study, dealing with the pozzolanic activity of isostructural sodium FAU zeolites with different framework compositions, together with those of the previous investigation on isochemical sodium zeolites with different structures (LTA and FAU) [11], outline an overall picture of the basic reactivity of this family of materials with regard to lime, giving useful indications on their use in manufacturing pozzolanic cements.

The kinetics of the hydration process, monitored up to 90 days, were successfully modelled with a pseudo-second-order reaction equation. The results showed that:

- During the first few hours, the main active factors are the Si/Al ratio (more precisely, the aluminium content) and the ion exchange capacity of the participating zeolite: both factors, which are in fact correlated, increase the amount of calcium removed from the contacting solution. Accordingly, zeolites L and X were able to subtract more calcium than Y zeolite, even if the concurrent effect of their smaller particle size could also have played a role.
- As the reaction proceeds, the specific surface and the silicon content of the zeolite become the dominant factor governing the reaction kinetics: the L zeolite fixed the lower lime amount (30%) in the shortest time (3 days), while the Y zeolite fixed about 47% of the lime in 90 days.
- Concerning the reaction products, while all three systems developed calcium aluminium oxide carbonate hydrate, which formed at first as a reaction product between zeolite and lime, at longer reaction times, the more aluminium-rich zeolites showed the presence of paraalumohydrocalcite, while the silicon-rich Y zeolite produced tobermorite as a reaction product.

Zeolite X (FAU) has demonstrated, in this study, the best overall pozzolanicity, because it couples reasonable reaction kinetics with a relevant ability to fix lime, which means that it also has a good capacity to increment the mechanical resistances of the hardened mortars.

## Figures and Tables

**Table 1:** Chemical composition (wt%) on a dry basis and some physical parameters of zeolite samples. Faujasite-type sodium zeolites: LSX, Si/Al ratio close to 1; X, Si/Al ratio close to 1.25; and Y, Si/Al ratio close to 2.5–2.8, L, obtained product.

Oxides	Samples			
Parameters	LSX ^1^	L	X	Y
SiO_2_	41.73	40.54	48.36	66.59
Al_2_O_3_	35.11	36.84	32.49	20.88
Na_2_O	15.67	21.36	19.15	12.52
K_2_O	7.48	1.25	–	–
Si/Al (mole)	1.01	0.93	1.25	2.71
(Na + K)/Al (mole)	0.97	0.97	0.97	0.98
Cell parameter, Å	25.011	25.057	24.948	24.651
BET (m^2^/g)	488	n.a. ^2^	408	669

^1^ As synthesized. ^2^ Not available.

**Table 2:** Grain size analysis of the four zeolite samples.

Sample	Grain Size Range (μm)	Average (μm)	Median (μm)
LSX	0.70–64.37	18.73	3.37
L	0.31–1.25	0.63	0.60
X	0.43–3.40	1.55	1.62
Y	0.38–70.85	26.91	25.17

**Table 3:** Analytical data of the Fratini test [27] (see also Figure 4).

Zeolite	[OH] mmol/l	[CaO] mmol/l	[CaO]_eq_ mmol/l	Undersaturation Degree, % *
L	215.3	1.18	1.75	32.5
X	220.9	0.33	1.70	80.6
Y	138.8	0.45	2.83	84.1

^*^ $\frac{{\left[CaO\right]}_{eq}-\left[CaO\right]}{{\left[CaO\right]}_{eq}}\times 100$

**Table 4:** Fixed Ca(OH)_2_ (% on weight) at various times for the zeolite–lime–water systems examined.

	Hours.	3	6	17	24	72	168	504	672	1344	2160
Zeo											
L		21.92	22.19	27.99	26.81	29.62	30.00	31.00	31.79	32.07	32.00
X		17.93	20.06	29.56	40.18	41.00	42.19	43.01	44.75	45.66	45.00
Y		7.76	10.59	14.06	22.56	33.24	44.75	45.66	46.00	46.50	46.67

**Table 5:** Pseudo-second-order kinetic parameters for the three investigated systems.

System	l_eq_, w% *	k, h^−1^	R^2^
Zeolite L–lime–water	30.9	1.92 × 10^–2^	0.981
Zeolite X–lime–water	44.7	3.75 × 10^–3^	0.978
Zeolite Y–lime–water	48.2	7.24 × 10^–4^	0.986

* l_eq_: equilibrium value of fixed lime; k: the rate constant of the reaction; R^2^: coefficient of determination.
